# Promoting labour migrant health equity through action on the structural determinants: A systematic review^☆^

**DOI:** 10.1016/j.jmh.2022.100082

**Published:** 2022-02-01

**Authors:** Mireille Evagora-Campbell, Aysha Zahidie, Kent Buse, Fauziah Rabbani, Sarah Hawkes

**Affiliations:** a Research Coordinator, Institute for Global Health, University College London, 30 Guilford Street, London, WC1N, UK; b Research Consultant, Aga Khan University, PO Box 3500, Stadium Road, Karachi, Pakistan; c Director, Healthier Societies Program, The George Institute for Global Health, Imperial College London, 84 Wood Lane, London, W12 0BZ, UK; d The Noordin M. Thobani Professor, Department of Community Health Sciences & Associate Vice Provost Research & Graduate Studies, Aga Khan University, PO Box 3500, Stadium Road, Karachi, Pakistan; e Professor of Global Public Health, Institute for Global Health, University College London, 30 Guilford Street, London, WC1N, UK

**Keywords:** Labour migrants, Structural drivers, Health inequity, Social determinants of health, Review

## Abstract

**Background:**

Labour migrants, who represent over sixty per cent of international migrants globally, frequently have poorer health status than the population of host countries. These health inequities are determined in a large part by structural drivers including political, commercial, economic, normative and social factors, including living and working conditions. Achieving health equity for migrant workers requires structural-level interventions to address these determinants.

**Methods:**

We undertook a systematic review of peer-reviewed literature designed to answer the question “what is the evidence for the effectiveness of interventions to address the structural determinants of health for labour migrants?” using the Ovid Medline electronic database.

**Findings:**

We found only two papers that evaluated structural interventions to improve the health of labour migrants. Both papers evaluated the impact of insurance – health or social. In contrast, we found 19 evaluations of more proximal, small-scale interventions focused on changing the knowledge, attitudes and behaviours of labour migrants.

**Interpretation:**

Despite the rise in international migration, including for work, and evidence that labour migrants have some higher health risks, there is a paucity of research addressing the structural determinants of health inequities in labour migrants. The research community (including funders and academic institutions) needs to pay greater attention to the structural determinants of health – which generally requires working across disciplines and sectors and thinking more politically about health and health inequities.

**Funding:**

Wellcome Trust (208712/Z/17/Z).

## 1 Introduction

Labour migrants represent over 60% of the 277 million international migrants globally^1^ whose lives are shaped by a range of structural and social factors that can lead to substantial health inequities compared with non-migrant populations. This paper adopts the ILO understanding of international labour migrants as “… all international migrants who are currently employed or unemployed and seeking employment in their present country of residence” (International Labour Organization 2017, International Labour Organization 2015). According to recent global estimates, approximately 58% of labour migrants are male (International Labour Organization 2021), with male migrants more likely than female migrants to work in low-income and lower middle-income countries (International Organization for Migration 2019). Migrant workers play a significant role in the global economy: in 2018, migrant workers globally remitted USD $689 billion – and in Nepal, Pakistan and Sri Lanka this income accounted for more than 8% of the country's GDP in 2020 (World Bank 2021).

The international import and export of labour on a mass scale, underpinned by the forces of globalisation and neoliberalism, and the absence of employment opportunities in home (often called ‘sending’) countries, reinforce a system of structural discrimination that leads to social inequalities and health inequities (Syed, 2016). However, the health concerns of this population are inadequately appreciated or understood and generally lacking attention in global public health research and response. A 2018 bibliometric analysis found that migrant workers were the focus of just 6.2% of published research on the health of migrants (Sweileh et al., 2018), which tends to be concentrated on high-income countries that receive migrants, rather than low- and middle-income countries (Wickramage et al., 2019). International organisations working to improve the health of migrants have tended to treat the domains of health and work separately, focusing either on infectious diseases and access to healthcare or on occupational health and safety, without recognising the important intersection between the two (Flynn and Wickramage, 2017).

### 1.1 Determinants of labour migrant health and health inequities

The health of labour migrants, like that of everyone, is determined by a range of societal drivers including those that are political, commercial, economic, environmental, normative, social and structural in nature. In this paper we focus on ‘upstream’ structural determinants^2^ which we consider to encompass both the social conditions in which people are born, grow, live, work, and age as well as the economic, commercial, legal and social policies, structures and institutions which drive these social conditions. Structural determinants, amongst which both migration and employment are key (Flynn and Wickramage, 2017), account for much of the health inequity between people and populations (Marmot et al., 2008). At the individual level, differences in social characteristics including age (Shao et al., 2016), class (O. Jubany and Castellanos, 2021), education attainment level (Peng et al., 2010), gender (O. Jubany and Castellanos, 2021), gender identity (Roche and Keith, 2014), income (Shao et al., 2016), marital status (Shao et al., 2016), migration status (International Labour Office 2010) and race (O. Jubany and Castellanos, 2021) have also been found to play a role in shaping migrant workers’ health outcomes.

Studies in several regions have documented the ‘healthy migrant effect’, or the better health status of migrants compared with the population of their origin country or the non-migrant population of the destination country (Helgesson et al., 2019). However, there are many dimensions of health where migrant workers may be at higher risks of ill-health due to social and structural inequalities. In many cases there is a shortage of empirical evidence on the health outcomes of migrant workers that has hindered our understanding of their health status and health needs – and may have undermined the ability to identify and implement appropriate national and international policy responses (Hargreaves et al., 2019).

Over a third of migrant workers are employed in industry (27%) – including manufacturing, construction, mining and quarrying – or agriculture (7%) (International Labour Organization 2021). Both sectors often involve long hours, hard physical labour and hazardous working conditions that increase the risk of occupational morbidities and accidents (International Organization for Migration 2021, Schenker, 2010). A 2019 systematic review and meta-analysis found that, amongst 7260 international migrant workers, 47% were estimated to have experienced at least one occupational morbidity and, amongst 3890, 22% had experienced a workplace injury or accident (Hargreaves et al., 2019). Male migrant workers are significantly more likely than female migrant workers to work in industry and agriculture (International Labour Organization 2021). A 2011 study of male migrant construction workers in India found that roughly 8% of the workers reported injury whilst at work (Laad et al., 2011) - with ‘unskilled’ workers significantly more likely to have poorer morbidity status than ‘skilled’ workers. In Singapore, fear of losing pay or being laid off was found to be a key reason that 15% of male migrant (mostly shipyard) workers intended to continue to work despite having a work-related injury (W. Lee et al., 2014).

As a result of prevailing gender norms and discrimination, women migrant workers, who represent 73% of all migrant domestic workers (United Nations General Assembly, 2019) – whose work is “performed in or for a household or households” (International Labour Organization, 2011) – may face heightened exposure to violence, exploitation and abuse in the workplace compared to male migrant workers (United Nations General Assembly, 2019). A 2014 study of 33 female migrant domestic workers who had been admitted to hospital in Lebanon found that 50% reported verbal abuse, roughly 38% physical abuse, and roughly 13% sexual assault - most of which was reportedly inflicted by employers (Zahreddine et al., 2014). In some countries, female workers are subject to pregnancy screening and to deportation if found to be pregnant (T. Loganathan et al., 2020). Limited access to sexual and reproductive health services – including due to fees for non-residents and those without medical insurance – (E. King and Dudina, 2019) fear of detention or deportation when seeking services (United Nations General Assembly, 2019), and an absence of linguistically and culturally appropriate health providers (International Organization for Migration, 2018) – particularly affect female migrant workers in some settings (E. King and Dudina, 2019).

Migrant workers may face elevated risk of communicable diseases due to social and work conditions (Guadagno, 2021). For example, the disproportionate number of COVID-19 cases and deaths documented amongst migrants (Hayward et al., 2021, Migration data portal 2021) may be a result of the high proportion of migrant workers in sectors with high exposure to COVID-19: more than 13% of all services and sales workers in seven of the twenty countries with the highest number of COVID-19 cases were foreign-born (Migration data portal 2021) and on average, 13% of all ‘key workers’ in the European Union (EU) are immigrants (Fasani and Mazza, 2020).

Despite improvements in international frameworks to protect and promote the rights of migrant workers since the Second World War, when there was an increase in migration within and from Europe (International Labour Office 2016) (see Table 1), the rights of labour migrants remain an issue of low priority on international human rights and development agendas. By October 2019, instruments designed to protect refugees or to combat people smuggling and human trafficking had been ratified by more than three quarters of UN Member States; in contrast, instruments protecting the rights of migrant workers had been ratified by fewer than 30% (International Labour Office 2016). Ratification of four key labour migration conventions remains low, particularly amongst high-income countries, which house the majority of labour migrants (Table 1) (International Labour Organization 2021). Infringements of internationally agreed protections for migrant workers in some countries persist, including being barred from joining trade unions or changing employer (Council on Foreign Relations 2021) and being excluded from some health services (T. Loganathan et al., 2020).

**Table 1:** Select international conventions on the rights of labour migrants.

Year adopted	Legal instrument	Ratification (High-income countries/total countries)
1949	ILO Convention concerning Migration for Employment (Revised), 1949 (No. 97)	17/51
1975	ILO Convention concerning Migrations in Abusive Conditions and the Promotion of Equality of Opportunity and Treatment of Migrant Workers (Supplementary Provisions), 1975 (No. 143)	7/26
1990	International Convention on the Protection of the Rights of All Migrant Workers and Members of Their Families	4/56
2011	ILO Convention concerning Decent Work for Domestic Workers, 2011 (No. 189).	10/32

From the start of his tenure, United Nations (UN) Secretary-General Antonio Guterres made regular migration a stated priority and in December 2018 the Global Compact for Safe, Orderly and Regular Migration, which contains commitments to migrants’ access to health, was endorsed by a large majority of UN Member States (United Nations 2018) which collectively hosted around 67% (181 million) of international migrants globally in 2019 (T. Loganathan et al., 2020).

Despite these potential enablers of increased multilateral engagement on health equity for migrant workers, a focus on the structural determinants of health is seldom applied to migrant populations (Castañeda et al., 2015). Moreover, despite evidence that migrant workers are at risk of poor health outcomes on account of the environments in which they live and work, there is limited literature examining the structural determinants of health of this population (Flynn and Wickramage, 2017, Castañeda et al., 2015).

The aim of this study is to examine the extent to which measures to address structural determinants as a means of improving labour migrant health are recognised, understood, evaluated and attended to in public health research.

## 2 Methods

We undertook a systematic review to answer the question “what is the evidence for the effectiveness of interventions to address the structural determinants of health for labour migrants?”. We searched the Ovid Medline electronic database for articles published in English at any date before July 2021, supplemented through hand-searching of references from included studies. Search terms are outlined in Table 2.

**Table 2:** Ovid Medline search terms.

Terms relating to migrant workers	Terms relating to structural determinants of health	Terms relating to interventions to address determinants of health	Terms relating to evaluation of the interventions	Search results excluded
• Employment/ • "Transients and Migrants"/ • Exp Human migration/ • Exp “Emigrants and immigrants/ • ((migrant? or migrat* or outmigrat* or immigra* or emigra* or non-national or foreign or overseas or expatriate or transient*) adj1 (work* or labo?r* or employ*)).ti,ab,kf. • ((migrant* or immigrant* or emigrant* or displaced or IDP or non-national or foreign or overseas or expatriate or transient*) adj1 (low skilled or blue collar or manual worker* or labo?rer* or construction worker* or construction labo?rer* or farmworker*)).ti,ab,kf. • (SML or single male labo?rer* or single male migrant* or guest worker* or economic migra*).ti,ab,kf.	• "Social Determinants of Health"/ • (social determinant* or structural determinant*).ti,ab,kf. • exp Socioeconomic Factors/ • Social Marginalization • (socioeconomic? or socio-economic? or economic status or poverty or education* or employment or unemployment or income).ti,ab,kf. • (social adj (status or factor? or conditions or marginali* or environment or disadvantage* inequalit* or disparit* or equalit* or equit* or inequit*)).ti,ab,kf.	• (intervention* or program* or project? or trial*).ti,ab,kf.	• Program Evaluation/ • (intervention* or program* or project? or trial*).ti,ab,kf	• exp Animals/ not Humans

Articles were screened to identify those papers that met the following objectives:

1 Population: Article evaluates interventions relating to male or female, internal or external labour migrants.^3^
2 Intervention: Article evaluates interventions to improve health outcomes that target structural determinants of ill-health.^4^
3 Control: Article may or may not use a control group to evaluate the effectiveness of the intervention.
4 Outcome: Article evaluates the effectiveness of the intervention at improving health outcomes.^5^
5 Article is published in English.

We used a standardised data extraction form, based on Population, Intervention, Comparison, Outcome (PICO) criteria (Schardt et al., 2007), to collect and collate data. Data was systematically extracted on: author(s); publication year; country in which the study was performed; target population; sex of targeted population; evaluated intervention; health area targeted; comparison group (if applicable); observed outcome of intervention. Titles and abstracts were screened and analysed independently by two reviewers; analysis of the final set of full papers was conducted by the same two reviewers, and any discrepancies were resolved by a third reviewer.

**Fig. 1.**
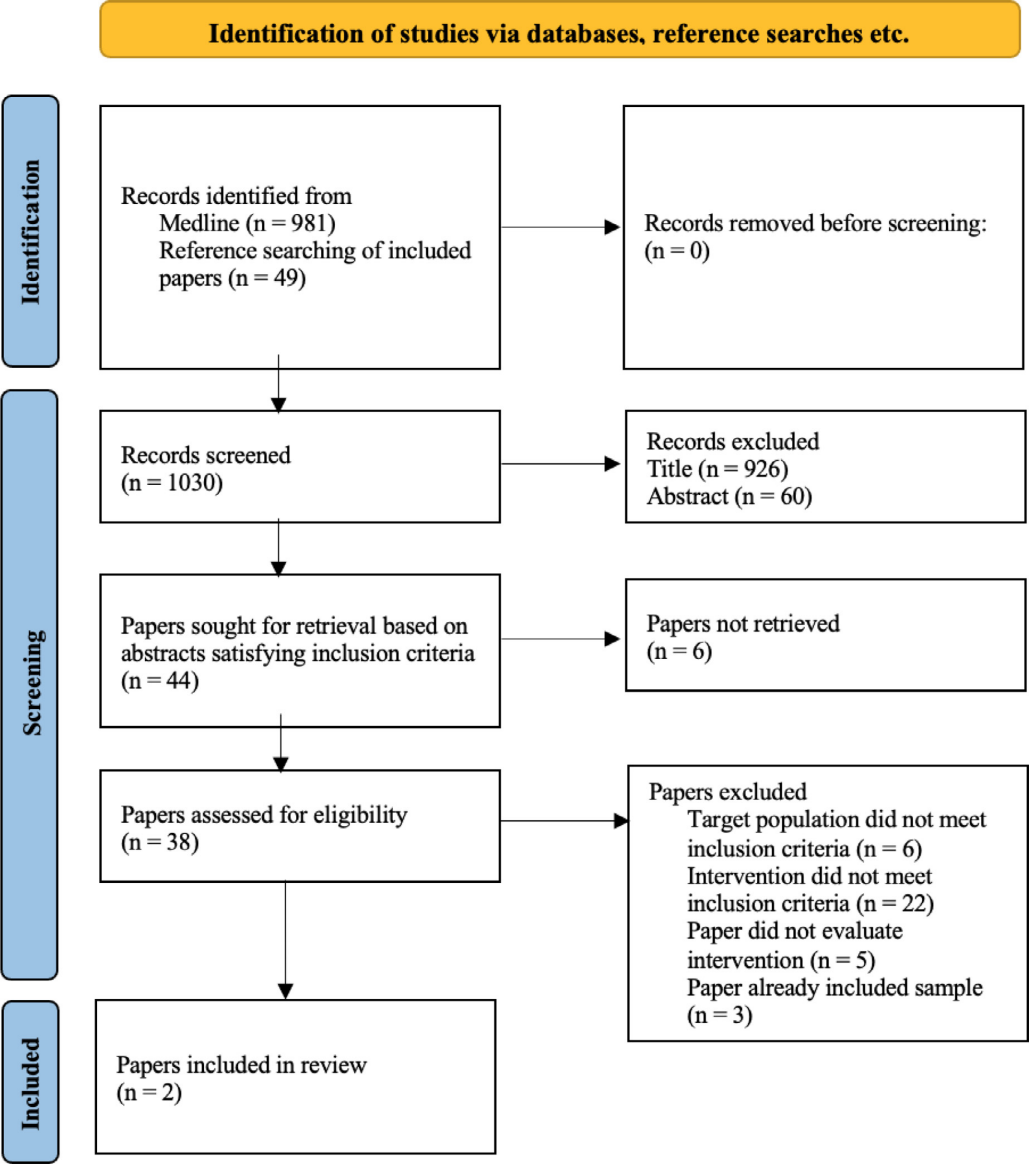
Results of review of literature evaluating the effectiveness of interventions to address structural determinants of migrant workers’ health.

Our initial screening identified 981 papers and, after exclusion of duplicates, we reviewed the abstracts of forty-six and the full text of twenty-seven papers - see Fig. 1. After application of our inclusion and exclusion criteria we identified two papers that evaluated structural determinants but an additional 19 papers addressing proximal determinants - i.e. interventions seeking to impact at the level of the knowledge, attitudes and behaviours of labour migrants, either individually or in groups. Two of these studies (Rodriguez et al., 2018, Qian et al., 2007) additionally sought to influence the behaviours of health care practitioners serving labour migrants.

Given the small number of papers meeting our inclusion criteria (i.e. 2 papers) we took the decision to also review the 19 papers that evaluated proximal interventions. For both sets of papers we used descriptive analytical methods only.

## 3 Results

We identified two studies evaluating interventions to address structural determinants both focused on financial interventions: health insurance and social insurance. Both reported on male and female internal migrants (i.e. Chinese workers, across a range of income categories, moving from rural to urban areas for work). The health insurance study (Zhang et al., 2020) compared migrants with insurance and those without and found that medical insurance: (i) significantly increased the likelihood that migrant workers would use health services; (ii) significantly decreased poor health outcomes; and (iii) “can statistically significantly improve the probability of migrant workers’ preventive medical service utilization”. The study further noted that women migrants were more likely to seek medical care and incur medical expenditures. The study on social insurance (Guan, 2019) compared self-rated health status amongst migrants with three kinds of insurance – unemployment, pension and workplace injury – against health status amongst uninsured migrants. All three types of social insurance were associated with higher levels of self-reported health status (i.e. higher likelihood of self-assessed health to be classified as “excellent” or “good”), although the effect was mediated by the presence of health insurance - see Table 3.

**Table 3:** Peer-reviewed articles identified in literature search addressing structural determinants of health - analysed according to PICO criteria.

Author and year of publication	Country where study was performed	Population targeted	Sex of population	Intervention	Health area targeted	Comparison group	Outcome
Guan 2019	China	Employed migrants in urban China	Female and male	Impact of three social insurance schemes (Unemployment Insurance, Pension Insurance and Workplace Injury Insurance) on Self-Rated Health Comparison (SRHC).	General health	Migrant workers with social insurance vs those without	All three social insurance schemes were associated with better SRHC (self-reported health comparison) (OR = 1.24, 95%CI: 1.02–1.51), (OR = 1.24, 95%CI: 1.07–1.45) and (OR = 1.72, 95%CI: 1.19–2.48) for UI (Unemployment Insurance), PI (Pension Insurance) and WII (Workplace Injury Insurance).
Zhang et al. 2020	China	Migrant workers in China	Female and male	The effects of health insurance on migrant workers’ utilisation of routine medical services, the medical burden, and the utilisation of preventive medical services	Utilisation of routine and preventative medical services and medical burden	N/A	Medical insurance significantly increased migrant workers’ probability of visiting a doctor, significantly reduced migrant workers’ medical burden and significantly improved the probability of preventive medical service utilization.

Amongst the 19 studies focused on proximal determinants, the population of focus was farmworkers (six studies), factory workers (5 studies), construction workers (three studies), and sex- or entertainment-workers (two studies). Three studies were not limited to a particular occupation. Seven of the studies were conducted in China, six in the USA and one in each of India, Indonesia and South Australia, Korea, Mexico and Guatemala, Singapore and Qatar. Of twelve studies that were targeted at a specific sex, eight were focused on female migrant workers, and four focused on males.

The majority of interventions evaluated by the studies were addressing sexual and reproductive health (10 studies) followed by occupational health and safety (4 studies), non-communicable diseases (4 studies) and hygiene (1 study).

Eighteen of the 19 studies used group-level interventions such as group counselling, peer-supported learning and educational programmes delivered to groups (e.g. through lectures or the use of audio-visual methods). One study (Suratman et al., 2016) examined an educational intervention which was delivered one-to-one. Nine interventions (Rodriguez et al., 2018, Qian et al., 2007, Chai et al., 2018, He et al., 2012, Hussain et al., 2020, Yang et al., 2017, Zhu et al., 2013, Zhu et al., 2014, Ning et al., 2013) supplemented group-level knowledge/awareness-raising with the distribution of educational materials such as leaflets and four studies (Qian et al., 2007, He et al., 2012, Zhu et al., 2014, Lim et al., 2018) also provided health products and services such as contraceptives or sexually transmitted infection (STI) screening and treatment services.

Nine studies (Guan, 2019, Chai et al., 2018, Yang et al., 2017, Zhu et al., 2014, Lim et al., 2018, Mitchell et al., 2015, Vela Acosta et al., 2005, Hovey et al., 2007, Kilanowski and Lin, 2013) compared outcomes amongst an intervention group and a non-intervention group. Five studies (He et al., 2012, Hussain et al., 2020, H. Lee et al., 2014, Ning et al., 2013, Shehadeh et al., 2018) compared outcomes of different interventions implemented amongst different groups of the study population. In four studies (Rodriguez et al., 2018, Ning et al., 2013, Kannappan and Shanmugam, 2019, Suratman et al., 2016) the comparison consisted of pre- and post-intervention measurements amongst the study population. One study (Febres-Cordero et al., 2018) used qualitative methods alone for assessing the impact of the intervention on HIV/STI prevention.

Of the 18 studies that reported quantitatively, all found the examined intervention(s) to have a statistically significant impact in at least one outcome measure, i.e. knowledge, attitudes, behaviours and/or health status indicator. The two studies which included interventions with health workers (Rodriguez et al., 2018, Qian et al., 2007), also showed a significant impact on levels of health knowledge amongst migrants, and also practice (increase in breast cancer awareness (Rodriguez et al., 2018, Qian et al., 2007) and contraceptive use (Qian et al., 2007)) – see Table 4.

**Table 4:** Peer-reviewed articles identified in literature search addressing non-structural determinants of health - analysed according to PICO criteria.

Author and year of publication	Country where study was performed	Population targeted	Sex of population	Intervention	Health area targeted	Comparison group	Outcome
Chai et al. 2018	China	Migrant workers in labour-intensive manufacturing factories	Female and male	5A group counselling regularly supported by social-media and traditional health education approaches	Knowledge of smoking and anti-smoking attitudes	Control group	The intervention arm improved smoking-related knowledge (OR = 2.40, 95% CI = 1.32–4.36, P = 0.02) and smoking-related attitude (OR = 3.07, 95% CI = 1.28–7.41, P = 0.03).
Cheng et al. 2011	China	Unmarried migrants working in construction sites in Chengdu, China	Male	Two intervention packages consisting of information about AIDS/STD prevention, free contraceptives, face-to-face counselling, peer education and hotlines	Sexual health	Two groups receiving each intervention package	Communication with others on sex increased in both groups: package B (25.6%); package A (9.3%). Both packages increased the use of condoms: package B (45.3%), package A (15.3%). Rate of unwanted pregnancy was significantly reduced in package B (OR = 0.318) but not in package A.
Febres-Cordero et al. 2018	Mexico and Guatemala	International migrant sex workers in the Mexico-Guatemala border	Female	Influence of peer support on migrant sex workers’ resilience related to social isolation, HIV/ STI prevention, and violence.	Sexual health/HIV	N/A	Qualitative results revealed that many women described peer support to be an important means for negotiating the challenges of social isolation and for HIV/STI prevention.
Hovey et al. 2007	USA	Migrant farmworkers in western Michigan	Female and male	Adolescent theatre program on HIV/AIDS knowledge and attitudes	Sexual health/ HIV	Control group	Participants reported significantly greater HIV/AIDS-related knowledge after viewing the performance (p < 0.01 to < 0.0001).
Hussain et al. 2018	Qatar	Migrant workers in a multinational construction project	Male	Four interventions to improve the transfer of acquired knowledge to the workplace: diverse learning stimuli; organisational support; development of self-efficacy, cross-cultural training.	Occupational health and safety	Three work groups with different training levels	The knowledge-transfer rate during the training session was affected by training levels and interventions (p< 0.05). Workers with additional interventions had better training transfer compared to the other groups (p< 0.05).
Kannappan et al. 2019	India	Factory workers	Female	Health education by peer education process on reproductive health issues focused on menstrual hygiene and reproductive tract infections, STIs including HIV and hepatitis B, family planning and cancers	Reproductive health	Pre- and post-intervention	A significant improvement in frequency of changing pads (P < 0.01), knowledge about family planning (P < 0.01), knowledge regarding both HIV and hepatitis B (P < 0.01) and knowledge of cervical cancer symptoms (P < 0.01) amongst the workers (P < 0.01) were observed after the intervention.
Kilanowski et al. 2013	USA	Latina migrant farmworker mothers in two Midwest States, USA	Female	Classes on nutrition knowledge, physical activity, healthy food choices	Nutrition and physical activity	Control group	The mean nutrition knowledge score amongst intervention mothers was significantly improved (t(18) = 2.97; P = 0.0082; paired t test).
Lee et al. 2014	Korea	Full-time Korean–Chinese migrant workers	Female	Stretching exercise interventions for musculoskeletal disorder (WMSD) education	Flexibility/ occupational health	Two groups receiving different Interventions	Significant increases in WMSD knowledge were noted at the 12 week assessment for both EI (P < 0.001) and SI (P = 0.013) groups. Social support had a significant increase in the EI group (t =-3.948,P < 0.001).
Lim et al. 2018	Singapore	Foreign Thai and Vietnamese entertainment workers	Female	Peer-led intervention consisting of HIV/STI education and condom negotiation skills, STI screening and treatment services and access to free condoms	HIV/STI prevention	Control group	The intervention group was more likely to report consistent condom use for vaginal sex with paid (aRR 1.77; 95% CI 1.71 to 1.83) and casual (aRR 1.81; 95% CI 1.71 to 1.91) partners than the comparison group. STI incidence was significantly lower in the intervention (6.8 per 100 FEWs) than the comparison (14.8 per 100 FEWs) group (aRR 0.42; 95% CI 0.32 to 0.55).
Mitchell et al. 2015	USA	Latinx farmworkers	Female and male	10-week adult education program on nutrition and exercise	Obesity prevention and reduction	Control group	Greater losses in weight, BMI, and waist circumference were associated with increasing attendance at intervention sessions (P values 0.0002, 0.0001, and 0.001, respectively).
Ning et al. 2013	China	Migrant workers in construction or mining sites in districts with high HIV prevalence	Male	Printed texts, audio-visual materials and expert- and volunteer-led discussions on benefits of male circumcision in reproductive and sexual health and HIV prevention	Sexual health/HIV	Three intervention groups: on-site session model, two-stage intervention model and three-stage model	Three-stage model was the most effective method to scale up MC, with RR = 2.0 (95% CI, 1.3–3.1, P=0.002) compared to the on-site session model.
Rodriguez et al. 2020	USA	Latinx immigrant farmworkers	Female and male	Training intervention for South Florida community health workers (CHWs) to educate Latinx immigrant farmworkers on breast cancer and early detection.	Breast cancer awareness	Pre- and post-intervention	All but one members who completed the rapid assessment survey stated that they learned something new about breast cancer. CHW self-reported evaluations also demonstrated this was an effective strategy to engage female Latino farmworkers in breast cancer education. ((p<0.01–0.001).
Shehadeh et al. 2017	USA	Alcohol and other drug-using migrant workers in South Florida	Female and male	An enhanced cognitive behavioural intervention and a health promotion control program for producing long-term reductions in HIV risk	Sexual health/HIV	Two groups receiving each intervention	At follow-up, participants reported an average of 1.46 (SD = 6.387) sex partners vs 2.14 (SD = 4.32) at baseline, 0.29 (SD = 0.83) unprotected sex partners vs 0.75 (SD = 1.70) at baseline and an average of 1.94 (SD = 5.32) unprotected vaginal sex acts vs 3.36 (SD = 6.97) at baseline.
Suratman et al. 2016	Indonesia and South Australia	Migrant farmworkers	Male	Educational intervention to improve knowledge and perceptions for reducing organophosphate exposure delivered one-on-one.	Occupational health and safety	Pre- and post-intervention	Knowledge about adverse effects of OPs was the only variable that was significantly improved amongst SA migrant farmworkers (P<0.001).
Qian et al. 2007	China	Female migrant factory workers (aged 16–30 years)	Female	Effective contraceptive use intervention including training factory doctors in family planning service delivery, lectures, educational materials, knowledge quiz, free contraceptives and a counselling service	Reproductive health	Control group	In the intervention group, reproductive health knowledge score increased significantly from 17.50 to 38.13 (p = 0.000) while women who had ever had sex in the last 3 months, contraceptive use increased from 70% to 93% and condom use increased significantly from 41% at baseline to 70%.
Vela Acosta et al. 2005	USA	Hispanic farmworkers in the USA	Female and male	A 60 min. pesticide training program about risks and first aid	Pesticide workplace health and safety	Control group	Program effectively increased farmworker's pesticide knowledge (P = 0.0001), SRP (P = 0.0001), and two (out of four) behaviour outcomes.
Yang et al. 2016	China	Migrant workers in Beijing City, China	Female and male	A combination of tailored print and video (TPV) and peer education on improving hand-washing skills	Hygiene	Control group	62.4% in the intervention group could wash their hands in a completely correct manner, compared to 23.8% in the control group (p < 0.05). The proportion of those who wash hands before eating every time in the intervention group increased significantly (88.6% vs 49.9% at baseline) (p < 0.05).
Zhu et al. 2014	China	Single, young (15–29) manual (factory) labourers	Female	Education materials, lectures about reproductive health. counselling classes and access to contraceptives. (Gynaecological care was provided when needed).	Reproductive health	Control group	The intervention cluster had a higher proportion of correct answers to queries about (HIV/AIDS) ((B) 0.047; P = 0.020) and awareness of places providing free contraceptives (odds ratio [OR] 2.011, 95% confidence interval [CI] 1.635–2.472; P < 0.001), while a significantly lower proportion accepted premarital sex (OR 0.492, 95% CI 0.416–0.582; P < 0.001), practicing premarital sex (OR 0.539, 95% CI 0.478–0.608; P < 0.001) or suffered from gynaecological disorders (OR 0.801, 95% CI 0.697–0.921; P = 0.002).
Zhu et al. 2013	China	Rural-to-urban migrant manual workers in a Chinese factory	Female	Educational materials and lectures about reproductive health, mental health and occupational health	Reproductive health, mental health, occupational health	Pre- and post-intervention	Participants reported higher General Health scores β = 0.056; P <0.001), Vitality scores (β = 0.066; P <0.001), Mental Health scores (β = 0.062; P <0.001), mental component summary scores (β = 0.040; P <0.001), and job satisfaction (OR) 2.104, 95% confidence interval [CI] 1.837–2.408; P <0.01).

Studies were not consistently reporting results disaggregated by socio-demographic variables. Three studies (Rodriguez et al., 2018, He et al., 2012, Febres-Cordero et al., 2018) contained recognition of gender as a determinant of health and, amongst the twelve studies in our sample that focused on a particular sex/gender in the population, four (He et al., 2012, Hussain et al., 2020, Ning et al., 2013, Suratman et al., 2016) examined interventions targeting male migrant workers. Seven of the twenty-one studies (Rodriguez et al., 2018, Zhang et al., 2020, Guan, 2019, Chai et al., 2018, Yang et al., 2017, Zhu et al., 2013, Ning et al., 2013) disaggregated their findings by a demographic characteristic other than sex: six examined the role of age (Zhang et al., 2020, Guan, 2019, Chai et al., 2018, Yang et al., 2017, Zhu et al., 2013, Ning et al., 2013), six education (Rodriguez et al., 2018, Zhang et al., 2020, Chai et al., 2018, Yang et al., 2017, Zhu et al., 2013, Ning et al., 2013), and four marital status (Guan, 2019, Chai et al., 2018, Yang et al., 2017, Zhu et al., 2013). Just two articles (Zhang et al., 2020, Chai et al., 2018) measured the impact of income-level on health outcomes and one each examined country of origin (Rodriguez et al., 2018), disability (Guan, 2019), ethnicity (Ning et al., 2013), native language (Rodriguez et al., 2018) or religion (Ning et al., 2013) – see Table 3.

## 4 Discussion

Labour migrants are seen by both sending and host countries as essential contributors to a globalised capitalist economy – with many high-income countries reliant on the import of labour for key functions in society and sending countries (or at least certain sections of societies) propped up by remittances. Yet inequities in their health outcomes, compared to host populations, highlight the health risks of this population. Many of these health inequities are driven by upstream structural and social determinants that place labour migrants, like many other types of migrant, in a position of vulnerability and marginalisation with respect to their legal status, living and working conditions, and power and status in host societies (Egli-Gany et al., 2020).

Addressing these structural determinants of health requires action by both governments and employers. Since these include determinants operating across the phases of the migration cycle - pre-departure, during travel, in the host community and upon return and reintegration (International Organization for Migration 2018) - actors in both origin and destination countries hold responsibility.

Within public health there has been a renewed call to expand action on the social determinants of health. For example, at the World Health Assembly, in May 2021, a resolution was adopted calling on Member States to “strengthen their efforts on addressing the social, economic and environmental determinants of health” (Seventy-Fourth World Health Assembly 2021, Buse, 2021). Despite such calls from the international community, coupled with commitments within the Global Compact (United Nations 2018), the public health research community appears to have paid relatively little attention to evaluating interventions to address structural determinants of labour migrant health. Our review, the first we know of, finds little in the way of rigorous empirical evidence on social and structural interventions. Instead, the literature focuses heavily on interventions targeting individual-level behaviours. This is despite evidence suggesting that changes in individual knowledge, attitudes and behaviours designed to lead to better health outcomes are facilitated within supportive social and structural environments (International Organization for Migration 2006, Castañeda et al., 2015, Flynn et al., 2015, Hanley et al., 2020).

The two papers from China targeting upstream determinants used insurance (health and social) as a means of improving access to health services and achieved significant improvements in health-care seeking as well as reported health status. In some settings, insurance schemes are now widely used to protect the health of migrants – including labour migrants. For example, in 2001 the Thai Ministry of Public Health introduced the migrant health insurance scheme for all migrants not covered by the national social health insurance scheme, to increase the affordability of public health facilities. These types of schemes can achieve high levels of population coverage. Between April and July 2016, almost 34% of the total estimated over 3400,000 migrant labourers in Thailand were enroled in the migrant health insurance scheme, a significant increase from less than 9% of who had been covered by the social health insurance scheme in 2011 (Tangcharoensathien et al., 2017).

In contrast, the individual or group-level interventions that comprised the majority of studies included in our review, although generally achieving a positive impact on measured outcomes (knowledge, attitudes, behaviour, individual health status), are less likely to reach a population at scale. Even when targeting at a proximal level, however, studies frequently did not take a more intersectional lens to understanding migrants and their health – data were presented with minimal disaggregation, thus reducing the capacity for analysis of the interaction of systems and structures of power and position and their impact on the lives of individuals.

The absence of rigorous evidence evaluating the impact of structural determinants on the health outcomes of labour migrants is a disappointing finding but may reflect a number of underlying challenges for research in this area. These include both the dearth of structural interventions to evaluate and well as difficulties accessing funding this type of research. In relation to the former, at least two considerations are at play. Firstly, despite research that has found that health is largely created outside the health sector (World Health Organization 2008), political attention and resources for improving population health continue to be predominantly invested in health care services rather than in the structural drivers of health – meaning that opportunities for evaluation are more limited. It has been proposed that this might be the case because structural interventions “threat[en] the social and economic status quo” and hence those with the power and resources to influence health policy (Newman et al., 2015). Similarly, the absence of political action on structural determinants might result from the disjuncture in time-frames: these interventions often take longer than bio-medical interventions and certainly longer than a typical political cycle, hence performance indicators and outcome measures are operating with different timelines across the relevant communities (Kelly et al., 2007).

In relation to challenges of research in this area, there are further considerations. Firstly, it might be the case that the bio-medical paradigm and worldview that dominates the health sector may result in the under-prioritisation of structural interventions by the influential research funding community (Kneipp et al., 2018). Secondly, while relationships between social factors and health are well established (Marmot and Wilkinson, 1999) (Solar and Irwin, 2007), the causal pathways linking the former with the latter are often long, complex and involving intervening factors (Link and Phelan, 1995), which can make establishing causal attribution of structural determinants on health outcomes challenging (Braveman and Gottlieb, 2014) (Kelly et al., 2007). The hierarchy of methodologies used in health research (Burns et al., 2011) may compound this as undertaking “high quality” research such as a randomised controlled trial (for example), would be highly unlikely in this field (Bharmal et al., 2015).

### 4.1 Limitations

While this study adopted a wide definition of structural determinants, it focused on a narrow population of interest, limiting the review to evidence on labour migrants. We recognise that in some settings, including in countries where a large proportion of migrants are migrant workers, interventions targeting the migrant population in its entirety may be effective at addressing the structural determinants of migrant worker health (Tangcharoensathien et al., 2017).

It is beyond the scope of this review to comment on the state of implementation by countries of the policy interventions evaluated in the literature. Development and implementation of national policies that align with the evidence base is dependent on the presence of formal and informal accountability mechanisms - including the adoption and ratification of international legally binding frameworks, non-binding multilateral agreements and an empowered migrant worker population that has adequate information on their rights and is engaged in policy-development processes.

Whilst our inclusion of a range of search terms and article types allowed us to work with a relatively large and diverse initial sample of results, the methodology would be strengthened by using a range of source databases, beyond Ovid Medline. Additionally, we only reviewed papers published in English, meaning that relevant literature published in other languages was excluded.

## 5 Conclusion

Our review of peer-reviewed literature on the effectiveness – i.e. health impact – of interventions addressing the structural determinants of the health of labour migrants has found little (2 papers) in the way of public health research. There is more evidence on proximal individual and group interventions targeting knowledge, attitudes and practice/behaviour. We believe this represents a significant gap in our understanding of what works to protect the health and reduce health inequities suffered by some of the most marginalised and least powerful people in the workforce. We therefore encourage the research community including funders and researchers as well as the people using evidence in policy and practice to collectively fill this evidence void. A collective approach to the co-production of knowledge, involving the collaboration of multiple and diverse stakeholder groups including the producers and users of knowledge, civil society, policy makers and policy influencers, is more likely to achieve knowledge uptake and utilisation (Hawkes et al., 2016). Moreover, such an approach is more likely to incorporate the lived experiences and views of the people whose interests and health status lie at the core of the issue – i.e. labour migrants.

In the context of global commitments to leave no one behind and achieve universal human rights, we cannot afford to let the health of labour migrants to be forgotten. Realising their rights will require understanding and addressing the structural determinants of health, beginning with building the evidence base for effective interventions.

## Declaration of Competing Interest

None.

## Footnotes

1 We use the terms ‘migrant worker’ and ‘labour migrant’ interchangeably in this paper.

2 In this paper we will use the term ‘structural determinants’ to encompass all upstream determinants of health - including those at the social level (Marmot et al., 2008).

## Footnotes

3 Where the target population of the intervention included both migrant workers and non-migrant workers, the article was only included where outcomes were disaggregated by each group.

4 Where the examined intervention contained multiple arms, some of which targeted structural determinants of health and some of which did not, the article was included only if the results of the arms were examined separately.

5 Articles needed not measure the impact of the intervention on health outcomes directly - they could measure the other outcomes such as health awareness, health seeking behaviours or uptake of infection mitigation behaviours.

## References

[bib0001] N. Bharmal K.P. Derose, M. Felician, and M.M. Weden, 2015. Understanding the Upstream Social Determinants of Health: Working Paper. RAND Health.

[bib0002] Braveman P., Gottlieb L. (2014). The social determinants of health: it's time to consider the causes of the causes. Public Health Rep..

[bib0003] Burns P.B., Rohrich J, Chung K.C. (2011). The levels of evidence and their role in evidence-based medicine. Plast. Reconstr. Surg..

[bib0004] Buse K. (2021). Healthy societies—fixing systems not people. BMJ Opin..

[bib0005] Castañeda H., Holmes S., Madrigal D., Young M., Beyeler N., Quesada J. (2015). Immigration as a Social Determinant of Health. Annu. Rev. Public Health.

[bib0006] Chai W., Zou G., Shi J. (2018). Evaluation of the effectiveness of a WHO-5A model based comprehensive tobacco control program among migrant workers in Guangdong, China: a pilot study. BMC Public Health.

[bib0007] Council on Foreign Relations. What Is the Kafala System? https://www.cfr.org/backgrounder/what-kafala-system (accessed 29 September 2021).

[bib0008] Egli-Gany D., Aftab W., Hawkes S., Abu-Raddad L., Buse K., Rabbani F. (2020). The social and structural determinants of sexual and reproductive health and rights in migrants and refugees: a systematic review of reviews. East Mediterr. Health J..

[bib0009] F. Fasani and J. Mazza, Immigrant Key Workers: Their Contribution to Europe's COVID-19 Response (unpublished paper) (2020).

[bib0010] Febres-Cordero B., Brouwer K.C., Rocha-Jimenez T., Fernandez-Casanueva C., Morales-Miranda S., Goldenberg S.M. (2018). Influence of peer support on HIV/STI prevention and safety amongst international migrant sex workers: A qualitative study at the Mexico-Guatemala border. PLoS One.

[bib0011] Flynn M.A., Wickramage K. (2017). Leveraging the domain of work to improve migrant health. Int. J. Environ. Res. Public Health.

[bib0012] Flynn M., Eggerth D., Jacobson C. (2015). Undocumented status as a social determinant of occupational safety and health: The workers’ perspective. Am. J. Ind. Med..

[bib0013] L. Guadagno, International Organization for Migration, Migrants and the COVID-19 pandemic: An initial analysis (2021).

[bib0014] Guan M. (2019). Associations between schemes of social insurance and self-rated health comparison: evidence from the employed migrants in urban China. Front. Public Health.

[bib0015] Hanley J., Larios L., Ricard-Guay A., Meloni F., Rousseau C. (2020). Pregnant and undocumented: taking work into account as a social determinant of health. Int. J. Migra. Health Soc. Care.

[bib0016] Hargreaves S. (2019). Occupational health outcomes among international migrant workers: a systematic review and meta-analysis. Lancet Glob. Health.

[bib0017] Hawkes S., Aulakh B.K., Jadeja N., Jimenez M., Buse K., Anwar I., Barge S., Odubanjo MO., Shukla A., Ghaffar A., Whitworth J. (2016). Strengthening capacity to apply health research evidence in policy making: experience from four countries. Health Policy Plan..

[bib0018] Hayward S. (2021). Clinical outcomes and risk factors for COVID-19 among migrant populations in high-income countries: a systematic review. J. Migra. Health.

[bib0019] He D., Cheng Y-M, Wu S-Z (2012). Promoting contraceptive use more effectively among unmarried male migrants in construction sites in China: a pilot intervention trial. Asia Pac. J. Public Health.

[bib0020] Helgesson M., Johansson B., Nordquist T., Vingård E., Svartengren M. (2019). Healthy migrant effect in the Swedish context: a register-based, longitudinal cohort study. BMJ Open.

[bib0021] Hovey J.D., Booker V., Seligman L.D. (2007). Using theatrical presentations as a means of disseminating knowledge of HIV/AIDS risk factors to migrant farmworkers: an evaluation of the effectiveness of the *Infórmate* program. J. Immigrant. Health.

[bib0022] Hussain R., Pedro A., Yeop Lee D., Chien Pham H., Sik Park C. (2020). Impact of safety training and interventions on training-transfer: targeting migrant construction workers. Int. J. Occup. Saf. Ergon..

[bib0023] International Labour Office, 2010. International Labour Migration: A rights-based approach. Geneva, ILO.

[bib0024] International Labour Office (2016). Promoting fair migration: International Labour Conference 105th Session 2016.

[bib0025] International Labour Organization, Convention concerning decent work for domestic workers 2011 (No. 189) (2011).

[bib0026] International Labour Organization, ILO global estimates on migrant workers: Results and Methodology (2015).

[bib0027] International Labour Organization, ILO Global Estimates on International Migrant Workers: Results and Methodology, Second Edition (2017).

[bib0028] International Labour Organization, ILO global estimates on migrant workers: Results and Methodology (2021).

[bib0029] International Organization for Migration, Migration Health Department, Migration: A Social Determinant of the Health of Migrants. Draft Background Paper (2006).

[bib0030] International Organization for Migration, Migration Health Division, The health of migrant workers & left-behind families (2018).

[bib0031] International Organization for Migration, IOM Global Migration Report 2020 (2019)

[bib0032] International Organization for Migration. Health of Labour Migrants. https://www.iom.int/health-labour-migrants, n.d. (accessed: 07 October 2021 ).

[bib0033] Jubany O., Castellanos R.Lázaro (2021). The Gender and Racial Construction of the Working Class: Temporary Mobility of Mexican Women Workers to the US and Canada. Gender Iss..

[bib0034] Jubany O., Castellanos R.Lázaro (2021). The Gender and Racial Construction of the Working Class: Temporary Mobility of Mexican Women Workers to the US and Canada. Gender Iss..

[bib0035] Jubany O., Castellanos R.Lázaro (2021). The Gender and Racial Construction of the Working Class: Temporary Mobility of Mexican Women Workers to the US and Canada. Gender Iss..

[bib0036] Kannappan S., Shanmugam K. (2019). Peer educators as change leaders – Effectiveness of peer education process in creating awareness on reproductive health among women workers in textile industry. Indian J. Commun. Med..

[bib0037] Kelly M.P., Morgan A., Bonnefoy J., Butt J., Bergman V. (2007).

[bib0038] Kilanowski J.F., Lin L. (2013). Effects of a healthy eating intervention on Latina migrant farmworker mothers. Fam. Commun. Health.

[bib0039] King E., Dudina V. (2019). The health needs of female labor migrants from Central Asia in Russia. J. Immig. Minor. Health.

[bib0040] King E., Dudina V. (2019). The health needs of female labor migrants from Central Asia in Russia. J. Immig. Minor. Health.

[bib0041] Kneipp S.M., Schwartz, Todd A., Drevdahl D.J., Canales M.K., Santacroce S., Santos H.P., Anderson R. (2018). Trends in health disparities, health inequity, and social determinants of Health Research A 17-Year Analysis of NINR, NCI, NHLBI, and NIMHD Funding. Nurs. Res..

[bib0042] Laad P., Chaturvedi R., Adsul B., Howal P. (2011). Health problems among migrant construction workers: a unique public-private partnership project. Indian J. Occup. Environ. Med..

[bib0043] Lee H., Chae D., Wilbur J., Miller A., Lee K L., Jin H. (2014). Effects of a 12 week self-managed stretching program among Korean-Chinese female migrant workers in Korea: a randomized trial. Jpn. J. Nurs. Sci..

[bib0044] Lee W. (2014). Health-seeking behaviour of male foreign migrant workers living in a dormitory in Singapore. BMC Health Serv. Res..

[bib0045] Lim R.B.T., Cheung O.N.Y., Tai B.C. (2018). Efficacy of multicomponent culturally tailored HIV/STI prevention interventions targeting foreign female entertainment workers: a quasi-experimental trial. Sex. Transm. Infect..

[bib0046] Link BG, Phelan J. (1995). Social conditions as fundamental causes of disease. J. Health Soc. Behav..

[bib0047] Loganathan T., Chan Z., de Smalen A., Pocock N. (2020). Migrant women’s access to sexual and reproductive health services in Malaysia: a qualitative study. Int. J. Environ. Res. Public Health.

[bib0048] Loganathan T., Rui D., Pocock N. (2020). Healthcare for migrant workers in destination countries: a comparative qualitative study of China and Malaysia. BMJ Open.

[bib0049] Marmot M., Friel S., Bell R., Houweling T.AJ, Taylor S. (2008). Closing the gap in a generation: health equity through action on the social determinants of health. Lancet North Am. Ed..

[bib0050] Marmot M., Wilkinson R. (1999).

[bib0051] Migration data portal. Migration data relevant for the COVID-19 pandemic. https://www.migrationdataportal.org/themes/migration-data-relevant-covid-19-pandemic (accessed 29 September 2021).

[bib0052] Mitchell D.C., Andrews T., Schenker M.B. (2015). Pasos saludables: A pilot randomized intervention study to reduce obesity in an immigrant farmworker population. J. Occup. Environ. Med..

[bib0053] Newman L., Baum F., Javanparast S., O'Rourke K., Carlon L. (2015). Addressing social determinants of health inequities through settings: a rapid review. Health Promot. Int..

[bib0054] Ning C., Jiang J., Ye L., Yang X., Wei B., Deng W. (2013). Comparison of three intervention models for promoting circumcision among migrant workers in western china to reduce local sexual transmission of HIV. PLoS One.

[bib0055] Peng Y., Chang W., Zhou H., Hu H., Liang W. (2010). Factors associated with health-seeking behavior among migrant workers in Beijing, China. BMC Health Serv. Res..

[bib0056] Qian X., Smith H., Huang W. (2007). Promoting contraceptive use among unmarried female migrants in one factory in Shanghai: a pilot workplace intervention. BMC Health Serv. Res..

[bib0057] Roche K., Keith C. (2014). How stigma affects healthcare access for transgender sex workers. Br. J. Nurs..

[bib0058] Rodriguez A., Hagevoort G.Robert, Leal D., Pompeii L., Douphrate D.I. (2018). Using mobile technology to increase safety awareness among dairy workers in the United States. J. Agromed..

[bib0059] Schardt C., Adams M., Owens T., Keitz S., Fontelo P. (2007). Utilization of the PICO framework to improve searching PubMed for clinical questions. BMC Med. Inf. Decis. Making.

[bib0060] Schenker M. (2010). A global perspective of migration and occupational health. Am. J. Ind. Med..

[bib0061] Seventy-Fourth World Health Assembly, Social determinants of health WHA74.16 (2021).

[bib0062] Shao C., Meng X., Cui S., Wang J., Li C. (2016). Income-related health inequality of migrant workers in China and its decomposition: an analysis based on the 2012 China Labor-force Dynamics Survey data. J. Chin. Med. Assoc..

[bib0063] Shehadeh N., Rubens M., Attonito J. (2018). Social support and its impact on ethnic identity and HIV risk among migrant workers. J. Rac. Ethn. Health Disparit..

[bib0064] Solar O., Irwin A. (2007).

[bib0065] Suratman S., Ross K., Babina K., Edwards J. (2016). The effectiveness of an educational intervention to improve knowledge and perceptions for reducing organophosphate pesticide exposure among Indonesian and South Australian migrant farmworkers. Risk Manag. Healthc Policy.

[bib0066] Sweileh W.M., Wickramage K., Pottie K. (2018). Bibliometric analysis of global migration health research in peer-reviewed literature (2000-2016). BMC Public Health.

[bib0067] Syed I.U. (2016). Labor exploitation and health inequities among market migrants: a political economy perspective. J. Int. Migra. Integr..

[bib0068] Tangcharoensathien V., Thwin A.A., Patcharanarumol W. (2017). Implementing health insurance for migrants, Thailand. Bull. World Health Organ..

[bib0070] United Nations, 2018. Towards a new global compact migration by António Guterres https://www.un.org/sg/en/content/sg/articles/2018-01-11/towards-new-global-compact-migration (accessed: 07 October 2021).

[bib0069] United Nations General Assembly (2019). Violence against women migrant workers.

[bib0071] Vela Acosta M.S., Chapman P., Bigelow P.L., Kennedy C., Buchan R.M. (2005). Measuring success in a pesticide risk reduction program among migrant farmworkers in Colorado. Am. J. Ind. Med..

[bib0072] Wickramage K., Simpson P.J., Abbasi K. (2019). Improving the health of migrants. BMJ.

[bib0073] World Bank, Annual Remittances Data (updated as of May 2021) (2021).

[bib0074] World Health Organization, Commission on the Social Determinants of Health, Closing the gap in a generation: health equity through action on the social determinants of health. Final Report of the Commission on Social Determinants of Health (2008).

[bib0075] Yang C., Hu J., Tao M. (2017). Effectiveness of a multifaceted intervention on improving the hand-washing skills and behaviors of migrant workers in Beijing. Glob. Health Promot..

[bib0076] Zahreddine N., Hady R., Chammai R., Kazour F., Hachem D., Richa S. (2014). Psychiatric morbidity, phenomenology and management in hospitalized female foreign domestic workers in Lebanon. Commun. Ment. Health J..

[bib0077] Zhang F., Shi X., Zhou Y. (2020). The impact of health insurance on healthcare utilization by migrant workers in China. Int. J. Environ. Res. Public Health.

[bib0078] Zhu C., Geng Q., Chen L. (2014). Impact of an educational programme on reproductive health among young migrant female workers in Shenzhen, China: an intervention study. Int. J. Behav. Med.

[bib0079] Zhu C., Geng Q., Yang H., Chen L., Fu X., Jiang W. (2013). Quality of life in China rural-to-urban female migrant factory workers: a before-and-after study. Health Qual. Life Outcome..

